# Exploration of thyroglobulin as a biomarker of iodine status in iodine-sufficient and mildly iodine-deficient pregnant women

**DOI:** 10.1007/s00394-023-03131-x

**Published:** 2023-03-27

**Authors:** Mariana Dineva, Margaret P. Rayman, Deborah Levie, Sandra Hunziker, Mònica Guxens, Robin P. Peeters, Mario Murcia, Marisa Rebagliato, Amaia Irizar, Alba Jimeno-Romero, Jordi Sunyer, Tim I. M. Korevaar, Sarah C. Bath

**Affiliations:** 1 Department of Nutritional Sciences, Faculty of Health and Medical Sciences, University of Surrey, Guildford, GU2 7XH UK; 2 The Generation R Study Group, Erasmus University Medical Centre, Rotterdam, The Netherlands; 3 Department of Internal Medicine, Academic Centre for Thyroid Diseases, Erasmus University Medical Centre, Rotterdam, The Netherlands; 4 Department of Child and Adolescent Psychiatry/Psychology, Erasmus MC, University Medical Centre, Rotterdam, The Netherlands; 5 ISGlobal, Barcelona, Spain; 6 Universitat Pompeu Fabra, Barcelona, Spain; 7 Spanish Consortium for Research on Epidemiology and Public Health (CIBERESP), Instituto de Salud Carlos III, Madrid, Spain; 8 Human Nutrition Laboratory, Institute of Food, Nutrition, and Health, ETH Zurich, Zurich, Switzerland; 9 Servicio de Análisis de Sistemas de Información Sanitaria, Conselleria de Sanitat, Generalitat Valenciana, Valencia, Spain; 10 Epidemiology and Environmental Health Joint Research Unit, FISABIO-Universitat Jaume I-Universitat de València, Valencia, Spain; 11 Predepartamental Unit of Medicine, University Jaume I, Castelló, Spain; 12 BIODONOSTIA Health Research Institute, Donostia‑San Sebastián, Spain; 13 Department of Preventive Medicine and Public Health, Faculty of Medicine and Nursery, University of the Basque Country (UPV/EHU), Leioa, Spain; 14 Parc Salut Mar-IMIM, Barcelona, Spain

**Keywords:** Thyroglobulin, Iodine, Biomarkers, Pregnancy, INMA, Generation R

## Abstract

**Purpose:**

Urinary iodine-to-creatinine ratio (UI/Creat) reflects recent iodine intake but has limitations for assessing habitual intake. Thyroglobulin (Tg) concentration, which increases with thyroid size, appears to be an indicator of longer-term iodine status in children and adults, however, less is known in pregnancy. This study investigated the determinants of serum-Tg in pregnancy and its use as an iodine-status biomarker in settings of iodine-sufficiency and mild-to-moderate deficiency.

**Methods:**

Stored blood samples and existing data from pregnant women from the Netherlands-based Generation R (iodine-sufficient) and the Spain-based INMA (mildly-to-moderately iodine-deficient) cohorts were used. Serum-Tg and iodine status (as spot-urine UI/Creat) were measured at median 13 gestational weeks. Using regression models, maternal socio-demographics, diet and iodine-supplement use were investigated as determinants of serum-Tg, as well as the association between UI/Creat and serum-Tg.

**Results:**

Median serum-Tg was 11.1 ng/ml in Generation R (*n* = 3548) and 11.5 ng/ml in INMA *(n* = 1168). When using 150 µg/g threshold for iodine deficiency, serum-Tg was higher in women with UI/Creat < 150 vs ≥ 150 µg/g (Generation R, 12.0 vs 10.4 ng/ml, *P = *0.010; INMA, 12.8 vs 10.4 ng/ml, *P < *0.001); after confounder adjustment, serum-Tg was still higher when UI/Creat < 150 µg/g (regression coefficients: Generation R, *B* = 0.111, *P* = 0.050; INMA, *B* = 0.157, *P* = 0.010). Iodine-supplement use and milk intake were negatively associated with serum-Tg, whereas smoking was positively associated.

**Conclusion:**

The association between iodine status and serum-Tg was stronger in the iodine-deficient cohort, than in the iodine-sufficient cohort. Serum-Tg might be a complementary (to UI/Creat) biomarker of iodine status in pregnancy but further evidence is needed.

**Supplementary Information:**

The online version contains supplementary material available at 10.1007/s00394-023-03131-x.

## Introduction

It is well known that maternal iodine deficiency during pregnancy, amongst other things, can cause marked intellectual disability in the offspring [1]; however, there is still a clear need for biomarkers that provide information about the usual individual iodine intake/status. This would facilitate the assessment of iodine status in populations (i.e., public-health monitoring), as well as the study of the health consequences of sub-optimal iodine intake and of interventions/policies aimed at improving iodine intakes.

Currently, the assessment of iodine status in populations and groups is conducted by comparing the median urinary iodine concentration (UIC, µg/L) from spot-urine samples against pre-defined cut-offs [2, 3]. As more than 90% of the dietary iodine absorbed is eventually excreted in the urine, UIC reflects individual recent iodine intake (i.e., in the last 24–48 h) but provides limited information about individual iodine status (i.e., habitual intake) and thyroid function [4]. UIC is usually measured in a single spot-urine sample and, as a result of day-to-day differences in hydration status (i.e., urine volume) and iodine intake, it can be misleading when used to estimate iodine nutritional status for an individual [5–7]. Adjustment of UIC by urinary creatinine concentration [i.e., by using the urinary iodine-to-creatinine ratio (UI/Creat, µg/g)] can correct for differences in individual hydration status since studies have shown that UI/Creat better reflects the 24-h urinary iodine excretion (24-h UIE) (i.e., a proxy for individual daily iodine intake) and serum iodine concentration than UIC alone [5, 7–10]. However, to estimate individual iodine status with 20% precision, at least 14 repeated spot-UIC measures or ten measured/estimated 24-h UIE measures are required from an individual [5]. This is impractical and costly in large population studies and in clinical settings.

Thyroglobulin (Tg), a thyroid-specific glycoprotein which is the site of the synthesis of thyroid hormones, is a potential biomarker of iodine status [11]. Small amounts of Tg are normally released into the circulation when iodine intake is sufficient [4]. In iodine-deficient areas, Tg concentration is positively correlated with thyroid volume, suggesting that Tg is an indicator of thyroid stimulation [12]. There is increasing evidence that Tg is a marker of iodine status in children and adults, showing that its concentration increases both in iodine deficiency and excess. For instance, in a cross-sectional study of schoolchildren from 12 countries, Tg concentration followed a U-shaped curve, with the lowest Tg concentration (~ 13 ng/ml) when median UIC was 100–299 µg/L (adequate and more-than-adequate iodine intake) [13]. In a cohort study of adults in three regions of China, the 5-years change (increase) in Tg was greater in the regions with mild iodine deficiency and excess than in the region with more-than-adequate iodine intake [14], though the difference was small.

In contrast to UIC, Tg concentration is thought to reflect iodine intake over a longer period (e.g., weeks to months) [4]. A randomised controlled trial (RCT) in mildly iodine-deficient adults showed that Tg decreased by 27% in response to 24 weeks of iodine supplementation (median 20 ng/ml at baseline vs 13 ng/ml at 24 weeks) while it remained unchanged in the placebo group (16 vs 15 ng/ml) [15]. A recent RCT in iodine-deficient pregnant women also showed that iodine supplementation from 12 weeks gestation until 8 weeks post-partum resulted in a reduction in Tg by 27% between baseline and the third trimester (median 8.4 vs 6.1 ng/ml, respectively), whereas Tg increased by 17% during the same period in the placebo group (8.3 vs 9.8 ng/ml); the cross-sectional differences in Tg between iodine *vs* placebo group were statistically significant in the third trimester but not in the second trimester or post-partum [16]. Secondary analyses of another RCT in mildly iodine-deficient pregnant women showed that supplementation from ≤ 14 weeks until delivery resulted in a lower Tg concentration in the iodine group in both second and third trimesters [17].

The usefulness of Tg as a biomarker of iodine status in pregnant women has not been widely explored in different settings of baseline iodine status. Since pregnant women can be particularly vulnerable to iodine deficiency [1], adequate assessment of the iodine status of this population group is important for both public-health monitoring and research.

This study, therefore, aimed to explore the usefulness of maternal serum Tg concentration (serum-Tg) as a biomarker of iodine status in pregnancy in settings of population iodine sufficiency (median UIC ≥ 150 µg/L) and mild-to-moderate iodine deficiency (median UIC 50–149 µg/L). The objectives of the study were to: (i) investigate whether various maternal characteristics, including diet and iodine-supplement use, are determinants of serum-Tg and (ii) explore cross-sectional associations between iodine status (measured as spot-UI/Creat; continuously and by groups as < 150 vs ≥ 150 µg/g and < 100, 100–149, 150–249, ≥ 250 µg/g) and serum-Tg during pregnancy. It was hypothesised that UI/Creat would be negatively associated with serum-Tg.

## Methods

### Study population

Samples and data from pregnant women recruited as part of two prospective population-based birth cohorts were used: Generation R in the Netherlands [18] and INfancia y Medio Ambiente (INMA) in Spain [19]. These studies have been described in detail elsewhere [18, 19] but briefly, in Generation R, pregnant women residing in Rotterdam with an expected delivery date between April 2002 and January 2006 were enrolled, and in INMA, pregnant women were recruited in the period November 2003 to January 2008 from regions of Spain (in this study, from Sabadell and Gipuzkoa).

**Fig. 1:**
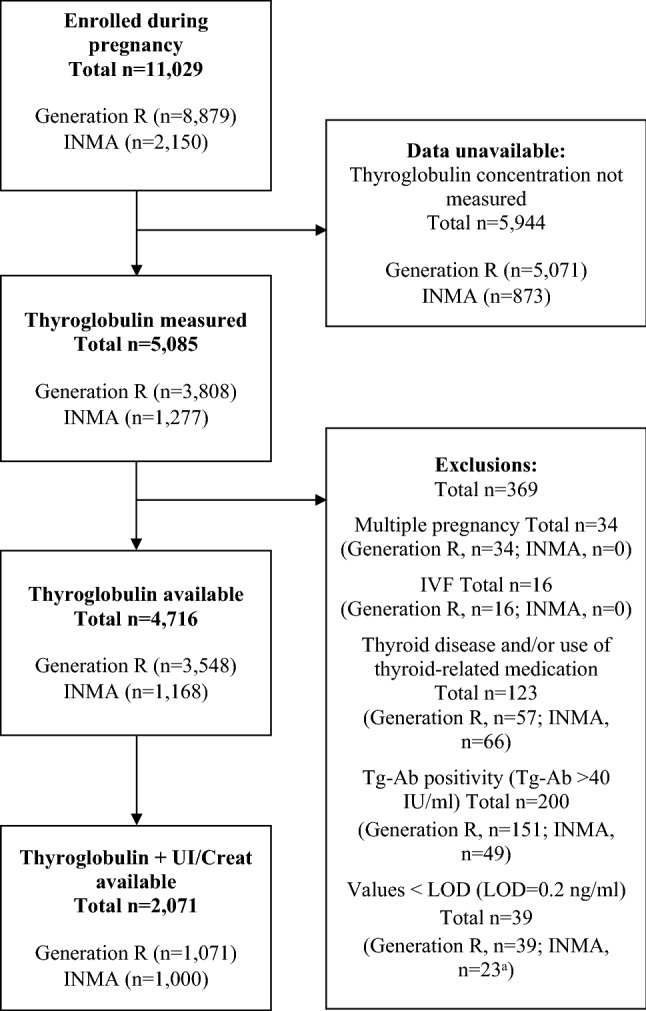
Flow chart of the study population selection ^a^In INMA, all values under the limit of detection (LOD = 0.2 ng/ml) were replaced with one half of LOD (0.1 ng/ml). In Generation R, the samples with values < LOD had been diluted three or six times prior to measurement due to insufficient sample volume, hence they were excluded from the analyses without replacing with half LOD (for full details see “Methods”). *IVF* in-vitro fertilisation, *LOD* limit of detection, *Tg-Ab* thyroglobulin antibody, *UI/Creat* urinary iodine-to-creatinine ratio

For the current study, women from both cohorts were eligible for inclusion if they had a serum-Tg measurement in pregnancy. Women with multiple pregnancies, in-vitro fertilisation (IVF), known thyroid disease, and/or use of thyroid-related medication were excluded (Fig. 1). Since the presence of thyroglobulin antibodies (Tg-Ab) can interfere with the measurement of Tg (i.e., Tg-Ab positive subjects can have underestimated or overestimated Tg depending on the assay used) [11], women with Tg-Ab titres above 40 IU/ml (manufacturer’s cut-off) were considered as Tg-Ab positive and were excluded.

Ethical approval for the cohorts had been obtained prior to recruitment from the Medical Ethical Committee of the Erasmus Medical Centre (Generation R; ethical approval number: MEC198.782/2001/31), the Ethical Committee of the Municipal Institute of Medical Investigation, and the ethical committees of the hospitals involved in the studies (INMA; ethical approval number: 2004/1851/I). All women gave informed consent.

### Serum Tg and Tg-Ab measurements

Blood samples in both cohorts had been collected after recruitment in early pregnancy [at median (25–75th percentile) 13.2 (12.2–14.9) weeks in Generation R and 13.4 (12.7–14.3) weeks in INMA]. Gestational week in both cohorts had been established using ultrasound examination. Serum Tg and Tg-Ab concentrations were measured at the ETH Zurich (Zurich, Switzerland) using an immunoassay calibrated using the manufacturer’s standards (IMMULITE, Siemens Healthcare Diagnostics, UK). The coefficients of variation (CVs) for serum-Tg were 9.3% at 1.9 ng/ml (*n* = 35), 7.1% at 9.6 ng/ml (*n* = 35) and 7.6% at 58.8 ng/ml (*n* = 35). The CVs for Tg-Ab were 8.1% at 38.5 IU/ml (*n* = 32) and 9.2% at 538.0 IU/ml (*n* = 32). According to the manufacturer’s cut-offs, serum-Tg > 55 ng/ml was considered elevated and Tg-Ab > 40 IU/ml was considered as positive. Data on thyroid peroxidase antibody (TPO-Ab) concentration (available only in Generation R) were also used. TPO-Ab concentration was measured using the Phadia 250 immunoassay; details are reported elsewhere [20]. Women were classified as TPO-Ab-positive if TPO-Ab measured more than 60 IU/ml, based on a previous study [20].

A small number of the measured serum-Tg values were under the assay limit of detection (LOD) (< 0.2 ng/ml) [1.0% (*n* = 39) in Generation R and 1.8% (*n* = 23) in INMA]. In Generation R, these values were from samples that had been diluted due to insufficient sample volume for analysis, and were excluded from statistical analyses. In INMA, samples had not been diluted and values below the LOD were replaced with one half of LOD (i.e., 0.1 ng/ml)—a common method for handling below-detection values [21]; such simple substitution techniques have been shown to produce little bias when the percentage of substituted values is low (i.e., 5–10%) [21]—in INMA, < 0.3% (*n* = 3) of all serum-Tg values were substituted.

### Urinary iodine and creatinine measurements

Spot-urine samples for the measurement of urinary iodine and creatinine concentrations had been collected at roughly the same time as the blood sample for serum-Tg measurement [median (25–75th percentile): 13.1 (12.1–14.6) weeks in Generation R and 13.4 (12.7–14.3) weeks in INMA]. The details of the laboratory measurement of urinary iodine and creatinine concentrations and the corresponding CVs are provided elsewhere for these cohorts [22]. To reduce the variability of UIC resulting from individual hydration-status differences, UIC was corrected by urinary creatinine concentration and the UI/Creat was used in preference to UIC [5, 10].

### Dietary assessment and iodine-supplement use

Maternal diet during pregnancy had been assessed using semi-quantitative food-frequency questionnaires (SFFQs) in early pregnancy (at the time when the blood and urine samples were collected). We have previously summarised the design of these SFFQs [23]. Comparable food groups to those in our previous study that investigated the determinants of UI/Creat in these cohorts were formed [23]. The data on food-group intake (g/day) were used to explore the association with maternal serum-Tg since some food-group intakes (e.g., milk and dairy products, fish and shellfish, and cereal and cereal products) were associated with UI/Creat in our previous study of these two cohorts [23].

Self-reported data on the use of iodine-containing supplements from pre-conception until enrolment in early pregnancy (approximately until the end of the first trimester) were available for all INMA women included in this study but only for a sub-set of the Generation R women [16.7% (*n* = 593)]. The iodine content of the supplements was available in INMA, but not in Generation R. Previous studies have shown that iodine supplementation in pregnancy can reduce maternal serum-Tg [16, 24–29]; in the current study, the iodine-supplement data were used to explore this in a mildly-to-moderately iodine-deficient and an iodine-sufficient population, i.e., INMA and Generation R, respectively.

### Statistical analyses

UIC, UI/Creat and serum-Tg were not normally distributed, therefore medians (25–75th percentiles) were reported. Women were dichotomised on the basis of having elevated serum-Tg; for the main analyses, this was defined as Tg > 40 ng/ml based on previous studies [11] and for sensitivity analyses, Tg > 55 ng/ml (the assay manufacturer’s cut-off) was used.

Differences in continuous variables (e.g., serum-Tg, UIC or UI/Creat) between groups based on maternal and dietary characteristics were explored using Mann–Whitney *U* and Kruskal–Wallis tests. Correlations were explored with Spearman’s rank correlation. Chi-square tests were used to compare the proportion of women with elevated serum-Tg across various categorical variables (e.g., by UI/Creat groups). Data on maternal characteristics (e.g., anthropometrics, parity, socio-demographic/economic factors, and lifestyle) had been collected in both cohorts and were recoded and harmonised as previously [23]. Multivariable regression models were used in each cohort with these maternal characteristics, as well as gestational week, and child’s sex as independent variables and serum-Tg as the dependent variable. Serum-Tg was transformed using the natural logarithm and the residuals of the models were visually assessed for normality to comply with parametric-test assumptions. Outliers were assessed by visual inspection of boxplots. Non-linearity of the associations of each continuous independent variable with serum-Tg was examined by adding their squared and cubic terms to the regression models, by plotting each potential determinant variable against serum-Tg and comparing the fit (*R*^2^) of a linear *vs* quadratic/cubic function through the data points.

Associations between maternal diet and serum-Tg were investigated using regression models with all estimated food-group intakes (g/day) as continuous predictors of serum-Tg (log-transformed) adjusted for estimated energy intake (kcal/day), gestational week, age, pre-pregnancy body mass index (BMI, kg/m^2^), TPO-Ab status (positive/negative; in Generation R only), ethnicity, parity, smoking status (never smoked, stopped smoking, continued smoking), alcohol consumption, education, net household income (in Generation R only), marital status (in Generation R only), living with a partner (in INMA only), and child’s sex. Effect sizes were expressed per 100-g intake, as previously described [23]. Food groups that are known good iodine sources were further explored as predictors of serum-Tg by grouping women in food-consumption categories using multiple linear [for the mean (log) Tg] and logistic [for the odds ratio (OR) of elevated Tg > 40 ng/ml and Tg > 55 ng/ml] regression models adjusted for the same covariates as above. The associations of iodine-supplement usage and daily iodine intake from supplements with serum-Tg were explored in multiple linear and logistic regression models adjusted for gestational week, age, pre-pregnancy BMI, TPO-Ab status, ethnicity, parity, smoking status, alcohol consumption, and education.

The continuous association between UI/Creat and serum-Tg (log-transformed) was explored using adjusted linear regression models. The selection of confounders that were included in all UI/Creat vs serum-Tg models was informed by (i) a previous study [30], (ii) the determinants of serum-Tg identified in this study (i.e., those identified in at least one of the cohorts) and (iii) the determinants of UI/Creat identified in our previous work [23]. The final models were adjusted for gestational week, age, pre-pregnancy BMI, ethnicity, parity, smoking status, alcohol consumption, education and TPO-Ab status (in Generation R only).

Women were split into groupings based on UI/Creat: (i) < 150 vs  ≥ 150 µg/g (reference group), and (ii) < 100, 100–149, 150–249 (reference group), ≥ 250 µg/g. The first grouping was based on WHO criteria for UIC that define adequate iodine intake in pregnant populations [2] and as used in other studies [30, 31]. The second grouping was based on previous Tg/thyroid studies [30, 32]. The association of these UI/Creat groupings with serum-Tg was explored in multiple regression models adjusted for the covariates.

Missing values on maternal characteristics were imputed for women included in these analyses using multiple imputation procedures as used and described previously [23]. Briefly, the automatic method in IBM SPSS Statistics was used, generating a total of 20 imputed datasets. Missing SFFQ data were not imputed due to wide inter-person variability. Details of the missing data that were imputed in each cohort are provided in Online Resource, Supplementary Table 1. All statistical analyses were conducted using IBM SPSS Statistics version 25.0 (IBM Corp., Armonk, NY, USA). Statistical significance was set at *P* < 0.05.

## Results

### Sample characteristics

After all exclusions, 4716 pregnant women had available data on serum-Tg: 3548 from Generation R and 1168 from INMA (Fig. 1). The characteristics of the study population by cohort are described in Table 1. Median serum-Tg during early pregnancy was 11.1 ng/ml in Generation R (3.6% > 40 ng/ml; at median 13.2 weeks) and 11.5 ng/ml in INMA (4.5% > 40 ng/ml; at median 13.4 weeks) (Table 2). According to the WHO median-UIC threshold for iodine sufficiency in pregnancy (median UIC ≥ 150 µg/L) [2], the group of pregnant women from Generation R (the Netherlands) was classified as iodine-sufficient in early pregnancy (median UIC: 166 µg/L at median 13.1 weeks; *n* = 1071), whereas the group from INMA (Spain) was mildly-to-moderately iodine-deficient (median UIC: 131 µg/L at median 13.4 weeks; *n* = 1000) (Table 2).

**Table 1:** Characteristics of the study population by cohort

Characteristics^a^	Generation R (*n* = 3548)	INMA (*n* = 1168)
**Maternal factors**		
Maternal age^b^ (years), mean (± SD)	29.7 (± 5.0)	31.5 (± 4.1)
Pre-pregnancy BMI (kg/m^2^), median (25–75th)	22.8 (20.7–25.7)	22.4 (20.7–24.8)
Positive TPO-Ab status, *n* (%)	130 (4.1%)	N/A
Ethnicity^c^, *n* (%)		
Reference group	1865 (52.5%)	1070 (91.6%)
Non-Dutch	1683 (47.5%)	N/A
Non-Spanish	N/A	98 (8.4%)
Parity, *n* (%)		
0	2033 (57.3%)	639 (54.7%)
1	1040 (29.3%)	446 (38.2%)
≥ 2	475 (13.4%)	83 (7.1%)
Smoking status, *n* (%)		
Never smoked	2,524 (71.1%)	836 (71.6%)
Stopped smoking	337 (9.5%)	160 (13.7%)
Continued smoking	687 (19.4%)	172 (14.7%)
Consuming alcohol, *n* (%)	1898 (53.5%)	109 (9.3%)
**Markers of socio-economic status**		
Education level, *n* (%)		
Low	386 (10.9%)	248 (21.2%)
Medium	1612 (45.4%)	464 (39.7%)
High	1550 (43.7%)	456 (39.1%)
Net household income (€ per month), *n* (%)		
Low < €1200	780 (22.0%)	N/A
Medium €1200–2000	713 (20.1%)	N/A
High > €2000	2055 (57.9%)	N/A
Marital status—married, *n* (%)	1703 (48.0%)	N/A
Living with a partner, *n *(%)	N/A	1157 (99.1%)
**Child factors**		
Child’s sex—male^d^, *n* (%)	1807 (50.9%)	587 (50.3%)

*BMI* body mass index, *N/A* data not available or not applicable, *SD* standard deviation, *TPO-Ab* thyroid peroxidase antibody

^a^Data presented as mean (± SD) for all continuous normally distributed variables, median (25–75th percentiles) for all continuous non-normally distributed variables, and *n* (%) for all categorical variables

^b^Maternal age at blood extraction

^c^Categories of ethnicity in Generation R (Reference group = Dutch, Non-Dutch = Indonesian, Cape Verdean, Moroccan, Dutch Antilles, Surinamese, Turkish, other Non-Western, Asian, and other Western) and in INMA (Reference group = Spanish, Non-Spanish = Latin-American and European/others); for a detailed breakdown of the numbers in each sub-category of the Non-Dutch and Non-Spanish ethnic groups, see Online Resource, Supplementary Table 2

^d^Data were not imputed for child’s sex in Generation R due to no missing values. Missing data on TPO-Ab were not imputed. The rest of the data are shown after multiple imputation of the missing values (see “Methods”).

**Table 2:** Descriptives of serum thyroglobulin and urinary iodine concentrations in Tg-Ab negative pregnant women by cohort

	*n*	Generation R (*n* = 3548)	*n*	INMA (*n* = 1168)
**Thyroglobulin**	3548		1168	
Gestational week at blood extraction^a^		13.2 (12.2–14.9)		13.4 (12.7–14.3)
Serum thyroglobulin (Tg), ng/ml^a^		11.1 (7.0–17.1)		11.5 (6.9–18.8)
Elevated Tg ( > 40 ng/ml), *n* (%)^b^		127 (3.6%)		52 (4.5%)
Elevated Tg ( > 55 ng/ml), *n* (%)^c^		58 (1.6%)		24 (2.1%)
**Iodine**	1071		1000	
Gestational week at urine sampling^a^		13.1 (12.1–14.6)		13.4 (12.7–14.3)
UIC, µg/L^a^		166 (97–285)		131 (76–222)
UI/Creat, µg/g^a^		209 (138–307)		147 (93–246)
UI/Creat < 150 µg/g, *n* (%)		311 (29.0%)		513 (51.3%)

*Tg* thyroglobulin, *Tg-Ab* thyroglobulin antibody, *UI*/*Creat* urinary iodine-to-creatinine ratio, *UIC* urinary iodine concentration

^a^Data presented as median (25–75th percentiles)

^b^Cut-off value for elevated Tg based on previous studies in adults and school-age children (see “Methods”)

^c^Cut-off value considered elevated from the laboratory where the Tg measurements were performed (i.e., assay manufacturer’s cut-off) (see “Methods”).

### Maternal characteristics, diet, and iodine-supplement use as determinants of serum-Tg

In both cohorts, women who reported smoking during pregnancy vs those who reported never smoking had a higher median serum-Tg (continued smoking vs never smoked: Generation R, 13.0 vs 10.7 ng/ml, *P* < 0.001; INMA, 15.8 vs 10.7 ng/ml, *P* < 0.001) (Online Resource, Supplementary Table 2). Smoking during pregnancy was still consistently positively associated with serum-Tg in analysis adjusted for other maternal characteristics (*P* < 0.001) (Online Resource, Supplementary Table 3). In INMA, even women who reported smoking only in the beginning of pregnancy and then stopped had a statistically significantly higher serum-Tg than women who reported never smoking [unstandardised regression coefficient *B* = 0.197, *P* = 0.034]. Smoking remained positively associated with serum-Tg after further adjustment for maternal diet and iodine-supplement use (data not shown).

Alcohol consumption was also associated with higher median serum-Tg in both cohorts (Online Resource, Supplementary Table 2), however, in adjusted analysis, this association was observed only in INMA (*B* = 0.205, *P* = 0.049) (Online Resource, Supplementary Table 3).

Ethnicity was associated with serum-Tg only in Generation R; compared to the Dutch women, Turkish women had lower serum-Tg (*B* = − 0.307, *P* < 0.001), whereas Cape Verdean (*B* = 0.164, *P* = 0.026) women and those from the Dutch Antilles (*B* = 0.404, *P* < 0.001) had higher serum-Tg (Online Resource, Supplementary Table 3). Adjustment for maternal diet did not substantially change the results, though serum-Tg of Cape Verdean women was not significantly higher (data not shown). Adjustment for overall supplement-use and iodine-containing supplement-use (in a smaller sample with available data) did not considerably change the results (data not shown).

In adjusted analysis, parity was negatively associated with serum-Tg only in Generation R (1 vs zero: *B* = − 0.082, *P* = 0.009; ≥ 2 vs zero: *B* = − 0.123, *P* = 0.007). In INMA, women in the medium or high education group had lower serum-Tg than those in the low education group (*B* = − 0.234, *P* = 0.002 and *B* = − 0.228, *P* = 0.004, respectively). Gestational week (at blood sampling), age, pre-pregnancy BMI, TPO-Ab status (available in Generation R only), household income, marital status/living with a partner and child’s sex were not associated with serum-Tg in the adjusted analyses (Online Resource, Supplementary Table 3).

In total, 2768 women from Generation R and 1155 women from INMA who had serum-Tg measures also had dietary data available. In INMA, there were no associations between intake of any food group (as continuous data) and serum-Tg in models adjusted for energy intake and other maternal characteristics (Online Resource, Supplementary Table 4). In Generation R, consumption of milk and dairy products was negatively associated with serum-Tg (*B*_(per100 g)_ = − 0.20, *P* = 0.030), whereas vegetable intake was positively associated with serum-Tg (*B*_(per100 g)_ = 0.58, *P* = 0.037); all other food groups were not significantly associated with serum-Tg (Online Resource, Supplementary Table 4).

When women were further categorised by just their milk intake (not including other dairy products due to insufficient numbers of non-consumers), INMA women who reported milk consumption had a lower median serum-Tg (unadjusted) than those who reported no consumption (Table 3). After adjustment for energy intake and socio-demographic/economic characteristics, compared to INMA women who consumed no milk, those who consumed ≤ 200 g/day or > 200 g/day had lower serum-Tg (*B*_(≤200 g)_ = − 0.328, *P* = 0.001; *B*_(>200 g)_ = − 0.269, *P* = 0.010). In adjusted logistic regression models, the odds of elevated serum-Tg (> 40 ng/ml) were lower in milk-consumers *vs* milk non-consumers (with a dose–response effect; Table 3). In Generation R, there was no significant difference in median serum-Tg between groups of milk intake (Table 3) and there was also no significant association in adjusted analyses [non-consumers (reference group): *B*_(≤200 g)_ = − 0.042, *P* = 0.534; *B*_(>200 g)_ = − 0.115, *P* = 0.102]. When looking at associations with elevated serum-Tg, a higher proportion of non-consumers had elevated Tg ( > 40 ng/ml, *P* = 0.045; Table 3), though this was not significant in the adjusted analysis (Table 3). The overall pattern of results between milk intake and elevated serum-Tg (but with a cut-off of 55 ng/ml) was similar in the sensitivity analysis for both INMA and Generation R, but the effect sizes were greater and the *P*-values of the associations were lower in the adjusted analyses (Online Resource, Supplementary Table 5).

**Table 3:** Median (25–75th percentiles) serum thyroglobulin concentration (serum-Tg, ng/ml), proportion (unadjusted) of pregnant women with elevated serum-Tg ( > 40 ng/ml^a^) and odds (adjusted) of elevated serum-Tg according to food-group intakes and iodine-containing supplement-use by cohort

	Generation R (*n* = 2768)							INMA *(n* = 1155)						
	Serum-Tg (ng/ml)			Elevated serum-Tg ( > 40 ng/ml) ^*a*^				Serum-Tg (ng/ml)			Elevated serum-Tg ( > 40 ng/ml) ^a^			
				Unadjusted		Adjusted					Unadjusted		Adjusted	
	Total *n*	Median (25–75th)	*P *^b^	*n* (%)	*P *^c^	OR (95% CI)	*P *^d^	Total *n*	Median (25–75th)	*P *^b^	*n* (%)	*P *^c^	OR (95% CI)	***P ***^***d***^
**Milk intake**			0.113		0.045^e^					0.004		0.097		
None	148	11.9 (7.3–18.0)		9 (6.1%)		Ref		111	14.1 (7.6–21.9)		9 (8.1%)		Ref	
≤ 1 glass (≤ 200 g/day)	1506	11.2 (7.2–17.4)		54 (3.6%)		0.762 (0.330, 1.761)	0.525	615	10.8 (6.2–18.0)		22 (3.6%)		0.360 (0.155, 0.836)	0.017
> 1 glass ( > 200 g/day)	1114	10.8 (6.8–16.6)		28 (2.5%)		0.511 (0.203, 1.285)	0.153	429	11.6 (7.9–19.0)		20 (4.7%)		0.347 (0.141, 0.855)	0.021
**Fish and shellfish intake**			0.093		0.014					0.199		1.000		
None (0 g/day)	555	10.4 (6.9–16.3)		28 (5.0%)		Ref		N/A	N/A		N/A		N/A	N/A
Some ( > 0 g/day)	2213	11.2 (7.1–17.3)		63 (2.8%)		0.604 (0.360, 1.013)	0.056	N/A	N/A		N/A		N/A	N/A
≤ 1 portion ( ≤ 120 g/day)	N/A	N/A		N/A		N/A	N/A	1041	11.6 (7.0–18.8)		46 (4.4%)		Ref	
> 1 portion ( > 120 g/day)	N/A	N/A		N/A		N/A	N/A	114	10.8 (6.1–18.4)		5 (4.4%)		0.912 (0.327, 2.539)	0.860
**Bread intake**			0.101		0.396					N/A		N/A		
≤ median ( ≤ 98 g/day)	1378	11.2 (7.1–17.9)		50 (3.6%)		Ref		N/A	N/A		N/A		N/A	N/A
> median ( > 98 g/day)	1377	10.9 (7.0–16.3)		41 (3.0%)		0.980 (0.569, 1.689)	0.942	N/A	N/A		N/A		N/A	N/A
**Salt intake** (inc**. **iodised)			N/A		N/A					0.186		0.361		
None (0 g/day)	N/A	N/A		N/A		N/A	N/A	374	12.0 (6.8–21.1)		20 (5.3%)		Ref	
Some ( > 0 g/day)	N/A	N/A		N/A		N/A	N/A	781	11.3 (6.9–18.1)		31 (4.0%)		0.683 (0.375, 1.242)	0.211
**Iodine-supplement** **use**^f^			0.476		N/A					0.005		0.017		
No	72	12.4 (8.1–15.2)		N/A		N/A	N/A	631	12.1 (7.3–20.1)		37 (5.9%)		Ref	
Yes	521	11.2 (7.6–16.2)		N/A		N/A	N/A	537	10.8 (6.4–17.3)		15 (2.8%)		0.524 (0.276, 0.997)	0.049

*BM*I body mass index, 95% *CI* confidence interval, *N/A* data not available or not applicable, *OR* odds ratio, *Ref* reference category, *serum-Tg *serum thyroglobulin concentration, *TPO-Ab* thyroid peroxidase antibody

^a^Cut-off value for elevated serum-Tg based on previous studies in adults and school-aged children (see “Methods”)

^b^*P*-values from a Mann–Whitney *U* test (for two-categorical variables) or a Kruskal–Wallis test (for variables with more than two categories)

^c^*P*-values from a Chi-square test (after Continuity Correction for comparisons in 2 × 2 tables)

^d^*P*-values from multiple logistic regression models with elevated serum-Tg as the dependent variable (categorised as > 40 ng/ml). In Generation R: (1) models with food-group intakes were adjusted for energy intake, gestational week at blood extraction, age, pre-pregnancy BMI, TPO-Ab status, ethnicity, parity, smoking status, alcohol consumption, education, net household income, marital status, and child’s sex; and (2) models with iodine-supplement use were not performed (see footnote ‘f’). In INMA: 1) models with food-group intakes were adjusted for energy intake, gestational week at blood extraction, age, pre-pregnancy BMI, ethnicity, parity, smoking status, alcohol consumption, education, living with a partner, and child’s sex; and 2) models with iodine-supplement use were adjusted for gestational week at blood extraction, age, pre-pregnancy BMI, ethnicity, parity, smoking status, alcohol consumption, and education

^e^Fisher's Exact Test *P*-value was reported due to some cells ( > 20%) with expected count < 5

^f^Data on iodine-supplement use were available only for a sub-sample in Generation R (see “Methods”) and statistical analyses with elevated serum-Tg (i.e., categorical outcome) were not performed in this cohort due to very small numbers; e.g., there was only 1 woman who was an iodine-supplement non-user and had elevated serum-Tg for the models with Tg > 40 ng/ml

Table 3 shows that there were mostly no differences in serum-Tg when splitting women by their intake of other known iodine sources [e.g., fish, bread, salt (including iodised salt)]. In Generation R, a lower proportion of women had elevated serum-Tg ( > 40 ng/ml) in the group who consumed some fish *vs* non-fish consumers (5.0 *vs* 2.8%; *P* = 0.014), though this association was attenuated in adjusted analyses (Table 3).

Iodine-containing supplements were taken by 46% (*n* = 537) of women in INMA; median serum-Tg was significantly lower in iodine-supplement users (Table 3), although this effect was not significant in adjusted analysis (*B* = − 0.045, *P* = 0.442). The mean (± SD) iodine intake from supplements in INMA was 38.6 (± 55.2) µg/day and only 7.5% (*n* = 88) had an iodine-supplement intake ≥ 150 µg/day. There was a weak negative correlation between the mean daily iodine intake from supplements and serum-Tg in INMA (*r*_s_ = − 0.089, *P* = 0.002, *n* = 1168), but this association did not remain in adjusted analyses (*B* = − 0.001, *P* = 0.144). A lower proportion of iodine-supplement users had serum-Tg > 40 ng/ml than non-users in INMA (2.8% vs 5.9%, *P* = 0.017; Table 3); this association was also observed in adjusted analyses (Table 3). In the sub-set of women in Generation R with data on supplement use (*n* = 593), 88% (*n* = 521) were iodine-supplement users (14.7% of the total Generation R sample from this study), but there was no significant difference in median serum-Tg in either unadjusted (Table 3) or adjusted analysis [users vs non-users (reference group): *B* = 0.023, *P* = 0.825].

### Association of single spot-UI/Creat with serum-Tg

Measurements of both serum-Tg and UI/Creat were available for 2071 women (Fig. 1). There was a weak negative correlation between UI/Creat and serum-Tg in Generation R (*r*_s_ = − 0.057, *P* = 0.064, *n* = 1071) and in INMA (*r*_s_ = − 0.150, *P* < 0.001; *n* = 1000). In analysis adjusted for confounders, UI/Creat as a continuous variable was not associated with serum-Tg in either cohort (Table 4). There was no evidence of a non-linear association between UI/Creat and serum-Tg (e.g., squared and cubic UI/Creat terms were not statistically significant and adding these terms to the models with serum-Tg did not improve the model fit; scatterplots also did not indicate non-linearity).

**Table 4:** Association^a^ between UI/Creat (continuous and as UI/Creat categories) and serum thyroglobulin concentration in pregnant women by cohort (adjusted analyses)

	Generation R (*n* = 1040)			INMA (*n* = 1000)		
	*n*	*B* (95% CI)	*P*^b^	*n*	*B* (95% CI)	*P*^c^
UI/Creat, µg/g	1040	− 0.000 (− 0.000, 0.000)	0.688	1000	− 0.000 (− 0.001, 0.000)	0.109
UI/Creat grouping 1						
< 150 µg/g	299	0.111 (0.000, 0.221)	0.050	513	0.157 (0.037, 0.276)	0.010
≥ 150 µg/g	741	Ref		487	Ref	
UI/Creat grouping 2						
< 100 µg/g	106	0.202 (0.023, 0.382)	0.027	284	0.206 (0.043, 0.370)	0.013
100–149 µg/g	193	0.082 (− 0.061, 0.225)	0.262	229	0.104 (− 0.067, 0.275)	0.232
150–249 µg/g	328	Ref		245	Ref	
≥ 250 µg/g	413	0.022 (− 0.096, 0.139)	0.718	242	0.005 (− 0.163, 0.174)	0.949

*BMI* body mass index, 95% *CI* confidence interval, *Ref* reference category, *Tg* thyroglobulin, *TPO-Ab *thyroid peroxidase antibody, *UI*/*Creat* urinary iodine-to-creatinine ratio

^a^Effect estimates (B = unstandardised regression coefficients), their 95% CIs and *P*-values are from multiple linear regression models performed for each cohort with (natural) log-transformed thyroglobulin (Tg) concentration as the dependent variable and urinary iodine-to-creatinine ratio (UI/Creat) continuous or UI/Creat groups as independent variables. Models were adjusted for maternal characteristics (for full models, see footnotes b and c). Reported B coefficients represent the change in the mean (natural) log of Tg (ng/ml) per unit increase in UI/Creat (µg/g) and for each UI/Creat category compared to the reference (‘iodine sufficient’) category

^b^Generation R models were adjusted for: gestational week at blood extraction, age (years), pre-pregnancy BMI (kg/m^2^), TPO-Ab status, ethnicity, parity, smoking status, alcohol consumption and education

^c^INMA models were adjusted for: gestational week at blood extraction, age (years), pre-pregnancy BMI (kg/m^2^), ethnicity, parity, smoking status, alcohol consumption and education

Women with UI/Creat < 150 µg/g had a statistically significantly higher median serum-Tg than those in the ≥ 150 µg/g group in both Generation R (12.0 vs 10.4 ng/ml, *P* = 0.010) and INMA (12.8 vs 10.4 ng/ml, *P* < 0.001) (Online Resource, Supplementary Table 6). In adjusted analyses, having UI/Creat < 150 µg/g vs ≥ 150 µg/g was associated with higher serum-Tg (Table 4).

**Fig. 2:**
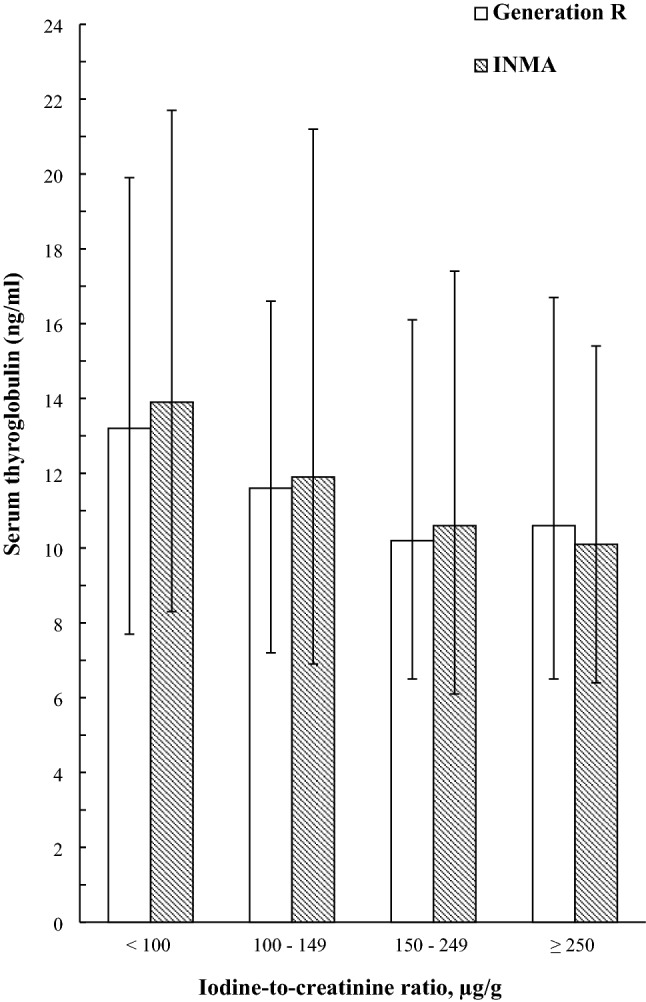
Median (25–75th percentiles) serum thyroglobulin concentration (Tg, ng/ml) according to categories of urinary iodine-to-creatinine ratio (UI/Creat, µg/g) in pregnant women from Generation R (white columns) and from INMA (shaded columns)

With four categories of UI/Creat ( < 100, 100–149, 150–249 and ≥ 250 µg/g), in both cohorts, women with UI/Creat < 100 µg/g had statistically significantly higher serum-Tg than the 150–249 µg/g group (i.e., reference group) (Fig. 2; Table 4; Online Resource, Supplementary Table 6). When looking at the group with UI/Creat < 50 µg/g, in INMA, the median serum-Tg was even higher at 14.4 ng/ml (*n* = 53) [this was not possible to report for Generation R due to small sample size (*n* = 7)].

There were no signs of higher serum-Tg in the groups of women with UI/Creat ≥ 250 µg/g (more-than-adequate iodine intake). Furthermore, when using a higher cut-off of ≥ 500 µg/g (excessive iodine intake), median serum-Tg was still not higher in either Generation R (10.0 ng/ml, *n* = 64) or in INMA (9.6 ng/ml, *n* = 53).

In INMA, UIC and UI/Creat were significantly lower in women with elevated serum-Tg ( > 40 ng/ml) than in women with serum-Tg in the ‘normal’ range (Table 5). Similar patterns were observed in Generation R but the differences were smaller and statistically non-significant. There was a higher proportion of INMA women with elevated serum-Tg in the group with UI/Creat < 150 µg/g than in the group with UI/Creat ≥ 150 µg/g (% with Tg > 40 ng/ml: 6.0% vs 2.7%, *P* = 0.014). The proportions of Generation R women with elevated serum-Tg were only slightly higher in the group with UI/Creat < 150 µg/g vs ≥ 150 µg/g and statistically non-significant (Table 5). The results of the sensitivity analyses using a cut-off of 55 ng/ml (the assay manufacturer’s cut-off) were similar (Online Resource, Supplementary Table 7).

**Table 5:** Descriptives of UIC and UI/Creat in pregnant women with “normal” vs elevated serum thyroglobulin concentration (Tg > 40 ng/ml) and the proportion with elevated Tg by UI/Creat groups in each cohort

Cut-off > 40 ng/ml^a^	Generation R (*n* = 1071)					INMA (*n* = 1000)				
	“Normal” Tg		Elevated Tg			“Normal” Tg		Elevated Tg		
	*n*	Tg ≤ 40 ng/ml	*n*	Tg > 40 ng/ml	*P*^b^	*n*	Tg ≤ 40 ng/ml	*n*	Tg > 40 ng/ml	*P*^b^
UIC, µg/L, median (25–75th percentiles)	1029	167 (98–286)	42	161 (97–256)	0.562	956	134 (77–225)	44	95 (63–138)	0.004
UI/Creat, µg/g, median (25–75th percentiles)	1029	210 (139–309)	42	174 (127–282)	0.082	956	148 (95–249)	44	105 (70–159)	0.005
***N (%)*** **with “normal” and elevated Tg**					0.651					0.014
UI/Creat < 150 µg/g	311	297 (95.5%)		14 (4.5%)		513	482 (94.0%)		31 (6.0%)	
UI/Creat ≥ 150 µg/g	760	732 (96.3%)		28 (3.7%)		487	474 (97.3%)		13 (2.7%)	

*Tg* thyroglobulin, *UI*/Creat urinary iodine-to-creatinine ratio, *UIC* urinary iodine concentration

^a ^Cut-off value for elevated Tg based on previous studies in adults and school-aged children (see “Methods”). For the results with a cut-off for elevated Tg > 55 ng/ml

(assay manufacturer’s cut-off), see Online Resource, Supplementary Table 7

^b^*P*-values from a Mann–Whitney *U* test or a Chi-square test (after Continuity Correction for comparisons in 2 × 2 tables)

### Discussion

This study aimed to identify the determinants of serum-Tg and explore its usefulness as a biomarker of iodine status in pregnant women. Several factors were associated with serum-Tg in pregnant women, including smoking, ethnicity, iodine-supplement use and milk intake. Furthermore, and as hypothesised, UI/Creat was negatively associated with serum-Tg in these two pregnancy cohorts from regions of sufficient and mildly insufficient iodine intakes; the association was most evident at group-level analyses but no continuous association was observed.

### Maternal characteristics, diet and iodine-supplement use as determinants of serum-Tg

As in several other studies in pregnant women [27, 33, 34], higher serum-Tg was found in smoking mothers in both pregnancy cohorts in our study. Smoking is a factor known to interfere with the function of the thyroid gland [35]. Thiocyanate, an end-product of tobacco smoke detoxification, can interfere with iodine transport in the thyroid by competitively inhibiting the sodium-iodide symporter (NIS), thereby decreasing iodide uptake by thyrocytes [36, 37]. Despite that inhibition, the increased thyroid activity from the autoregulatory mechanisms that maintain iodide uptake for thyroid-hormone synthesis, results in an increase in thyroid volume and serum-Tg; thus Tg is a non-specific marker of the activity or stimulation state of the thyroid [38, 39]. The effect of smoking on serum-Tg seemed stronger in INMA than in Generation R, perhaps because iodide transport by the NIS is affected more by competitive inhibition when iodine intake is lower [34, 38].

In Generation R, compared to Dutch women, Turkish women had significantly lower serum-Tg, whereas women from the Dutch Antilles had significantly higher serum-Tg. These differences could be, at least partly, explained by differences in iodine intake. In our previous study in this cohort, we also showed that, compared to Dutch women, Turkish women had significantly higher UI/Creat, whereas women from the Dutch Antilles had significantly lower UI/Creat [23]. Ethnicity was not associated with serum-Tg in INMA, though the INMA cohort was not as multi-ethnic, the proportion of non-Spanish ethnic groups being very small.

Pregnant women from INMA who used iodine-containing supplements before pregnancy and in the first trimester had statistically significantly lower serum-Tg, with a smaller proportion of this group having elevated serum-Tg ( > 40 ng/ml) than the group of non-users. In adjusted analysis, however, iodine-supplement use was not a significant predictor of maternal serum-Tg (continuous). INMA women were mildly-to-moderately iodine-deficient, and therefore, iodine supplementation might have alleviated the increase in Tg associated with thyroidal adaptation to low iodine intakes (i.e., enhanced thyroidal stimulation) [39]; though the difference in serum-Tg between women who used iodine supplements and those who were non-users was small (about 1 ng/ml) and probably clinically insignificant. Our recent systematic review on the effects of iodine supplements in mildly-to-moderately iodine-deficient pregnant women showed that most RCTs found that iodine supplementation resulted in a reduction in maternal serum-Tg by 18–37% over the course of pregnancy and cross-sectional evidence showed lower Tg in iodine-supplement users than in non-users (mean difference ranged from 5.3 to 11.1 ng/ml) [40]. In the current study, although there was a significant negative correlation between the mean daily iodine intake from supplements and serum-Tg in INMA women, it was only weak and the association was not significant in adjusted analyses, possibly suggesting that the dose of iodine used by most women might have been insufficient to have an effect on Tg [e.g., total mean daily iodine intake from supplements was < 40 µg/day and only 8% (*n* = 88) had an iodine-supplement intake ≥ 150 µg/day]. An intervention study in mildly-to-moderately iodine-deficient pregnant women showed that Tg decreased by 36% over the course of pregnancy in the group taking the higher dose of iodine supplement (200 µg/day), while it increased by 25% in the group with the lower dose (50 µg/day) [41]. It was not possible to fully explore the association of iodine supplements with Tg in Generation R because the data on iodine-supplement use were collected only for a limited number of women in this cohort and within this group, the number of women who did not use supplements (i.e., the reference group) was very small relatively to those who reported iodine-supplement use. It should, however, be noted that women in Generation R were overall iodine-sufficient so iodine-supplementation would not have been expected to prevent an increase in Tg that might be related to iodine deficiency.

Milk intake in pregnancy was associated with lower serum-Tg in both cohorts. In our previous study, milk and dairy-product intake was an important predictor of maternal UI/Creat in both of these cohorts [23]. Together these findings highlight the potential negative impact of plant-based diets/diets low in milk and dairy products on iodine intake. Although in our previous study the intake of cereal products (e.g., bread, which is fortified with iodine in the Netherlands) and fish were predictors of UI/Creat in Generation R and INMA, respectively, these food groups were not associated with serum-Tg in the respective cohorts in the current study. The lack of these associations with serum-Tg could be because most Generation R women consumed bread regularly (i.e., there was not enough variability in bread consumption and it was not possible to create a non-consumer group) and all INMA women consumed fish thus, the differences in intakes of these food groups between individuals might have not been sufficiently large to observe differences in Tg. There was no association between salt intake (including iodised salt) and serum-Tg in INMA; this could be because of the limitations of FFQs in quantifying total salt intake [4]. The observed positive association between vegetable intake and serum-Tg in Generation R is unexpected but vegetable intake could be a marker of a plant-based diet (generally low in iodine) and/or of goitrogen intake (inhibitors of iodine utilisation).

### Association of single spot-UI/Creat with serum-Tg

As hypothesised, UI/Creat was negatively associated with serum-Tg (in analyses at group level). In early pregnancy (at median ~ 13 weeks), women with UI/Creat < 150 µg/g had higher serum-Tg than women with UI/Creat ≥ 150 µg/g in both cohorts. Serum-Tg was even higher in the groups of women with UI/Creat < 100 µg/g and in INMA women with UI/Creat < 50 µg/g. The high serum-Tg in the groups with suboptimal iodine nutrition is likely a result of increased thyroid activity. The thyroid adapts in order to keep up with the increased demand for thyroid hormone during pregnancy, even though the iodine supply is insufficient, thereby leading to an increased amount of Tg that is being released in the circulation [39]. These results, therefore, suggest that Tg could be an indicator of iodine nutrition in groups of pregnant women.

A weak negative correlation was found between UI/Creat and serum-Tg, particularly in INMA women; though, this continuous association was not statistically significant in either cohort in adjusted analysis. This finding is in agreement with other studies that have investigated the continuous relationship of UI/Creat and UIC with serum-Tg during pregnancy [27, 32, 42, 43]. Unlike urinary iodine concentration which reflects recent iodine intake (i.e., in the last 24–48 h) and is not directly indicative of thyroid function, Tg concentration provides information about longer-term iodine intake (i.e., in weeks to months) [4] and is a non-specific marker of thyroid activity [44]. A strong association between the two would not be expected on an individual level (i.e., in continuous analyses), as iodine concentration in a casual spot-urine sample is an imprecise estimate of habitual iodine intake in an individual [5].

Although Generation R women were classified as iodine-sufficient and INMA women were classified as mildly-to-moderately iodine-deficient according to the median UIC, the overall median serum-Tg and the percentage of women with elevated serum-Tg were only slightly higher in INMA women. Most previous studies in iodine-deficient pregnant populations have reported a median Tg ≥ 13 ng/ml [11, 27, 30, 32]. In the current study, the overall median serum-Tg in both cohorts was around 11 ng/ml and it reached ≥ 13 ng/ml only in the sub-groups with UI/Creat < 100 µg/g. As serum-Tg was measured in the first trimester, it could be that women in INMA could draw on their iodine stores to compensate for their low iodine intake. With advancing pregnancy, however, if iodine intake remains suboptimal, the stores might get progressively depleted resulting in a progressive rise in serum-Tg concentration [39]. Indeed, a previous study in mildly-to-moderately iodine-deficient pregnant women has shown that Tg increased over the course of gestation in the group of women with UI/Creat < 150 µg/g but remained unchanged in the group with UI/Creat ≥ 150 µg/g [30]. This being the case, the differences in serum-Tg between the two cohorts in this study might have been more apparent if serum-Tg had been measured later in pregnancy. Only data on serum-Tg from early pregnancy (up to 18 weeks) were available for this study, thus it was not possible to explore the interaction between UI/Creat and a wider range of gestational week on serum-Tg.

In both cohorts, the median serum-Tg of pregnant women in the groups with sufficient iodine intake (i.e., UI/Creat ≥ 150 µg/g) was around 10 ng/ml. These results are in agreement with a recent study that measured Tg from a dried-blood spot in pregnant women across 11 countries and suggested that a median Tg ~ 10 ng/ml can be used to indicate iodine sufficiency in a pregnant population [43]. By contrast, several studies in non-pregnant adults have suggested a higher median Tg cut-off value (< 13 ng/ml) that indicates iodine sufficiency in a non-pregnant adult population [11, 15].

Previous studies in schoolchildren and pregnant women have shown a U-shaped association between UIC and Tg, where a higher Tg concentration was observed in the groups with low and excessive iodine intakes [13, 45]. Similar results were also reported in a study with data from 11 countries with varying iodine status, where Tg was higher in pregnant women residing in countries with severe iodine deficiency and more-than-adequate iodine intake than in those with sufficient intake [43]. In the current study, however, serum-Tg was not higher in the groups with more-than-adequate intake (UI/Creat ≥ 250 µg/g) when compared with the group with adequate intake. Additionally, there was no significant non-linear association between UI/Creat and serum-Tg. However, it should be noted that the number of women in the groups with excessive iodine intake in both cohorts was probably too small to detect these effects (in each cohort, < 65 women had UI/Creat ≥ 500 µg/g). Women in these UI/Creat groups might also not have had more-than-adequate/excessive iodine intake over a longer period (as this grouping was based on a single spot-UI/Creat) to result in an increased Tg concentration.

The negative correlation between UI/Creat and serum-Tg in women from Generation R was weak and the continuous association was not significant in adjusted analysis. In agreement with those results, a previous study with data from multiple countries found no statistically significant correlation between UIC and Tg in countries with sufficient or more-than-adequate iodine intake though a significant negative correlation was found in iodine-deficient countries [43]. Similar findings were reported in studies in iodine-sufficient pregnant women from China and Brazil [46, 47]. The difference in serum-Tg between the UI/Creat groups ( < 150 vs  ≥ 150 µg/g) was more subtle in Generation R than in INMA. Moreover, the proportion of Generation R women with elevated serum-Tg (Tg > 40 and > 55 ng/ml) was similar in the groups with UI/Creat < 150 µg/g and ≥ 150 µg/g. The median UI/Creat was also similar between the group of women with elevated serum-Tg (Tg > 40 and > 55 ng/ml) and the group with ‘normal’ serum-Tg; the median UI/Creat in both of these groups was indicative of sufficient iodine intake (i.e., UI/Creat ≥ 150 µg/g). Conversely, in INMA, UI/Creat was nearly 150 µg/g in the group of women with ‘normal’ serum-Tg and ~ 100 µg/g in the groups with elevated serum-Tg. The more subtle association between UI/Creat and serum-Tg in Generation R could be because most Generation R women had UI/Creat ≥ 150 µg/g (i.e., ~ 70%) thus, this cohort had limited variation in iodine intake. It is possible that, within populations that are overall iodine-sufficient, even if iodine intake becomes low during pregnancy (e.g., low UI/Creat at the time of urine-sampling), women might have good iodine stores (as they live in an iodine-sufficient country) to keep up with the increased thyroid-hormone production during pregnancy without stressing the thyroid, thereby preventing the increase in serum-Tg.

Although in these analyses there was an association of spot-UI/Creat with serum-Tg during pregnancy, it should be noted that Tg is a non-specific marker of iodine nutrition and is also dependent on thyroid activity [48]. The effect of pregnancy on thyroid activity should be considered when using Tg as a biomarker of iodine nutrition in pregnant women as physiological changes occur that are unrelated to iodine status and can influence Tg [e.g., an increased thyroid secretory activity and a peak in human chorionic gonadotropin (hCG) at the end of the first trimester] [42, 48].

Overall, our results demonstrated that Tg might be a marker of iodine status in groups of pregnant women (i.e., at group/population level), with higher serum-Tg in the groups of women with low iodine intakes (particularly when UI/Creat ≤ 100 µg/g). Median serum-Tg around 10 ng/ml seems indicative of sufficient iodine intake (defined by UI/Creat ≥ 150 µg/g) in a group of pregnant women, confirming the findings by Stinca et al*.* 2017 [43]. Tg concentration might be a more sensitive biomarker of iodine status in pregnant populations if measured later in pregnancy (e.g., in the second/third trimester; though these later measures of iodine status might not be as useful as measures earlier in pregnancy, considering the importance of sufficient iodine intake in the first trimester) for two reasons: (i) the ability of Tg to distinguish between an iodine-sufficient and mildly-to-moderately iodine-deficient pregnant population might be dependent on the maternal thyroid iodine stores; if iodine intake remains low in pregnancy, the stores will get progressively depleted resulting in a progressive rise in serum Tg in the later stages of pregnancy and a more apparent difference between an iodine-sufficient and a mildly-to-moderately iodine-deficient group of pregnant women; (ii) Tg is a non-specific marker of thyroid stimulation and thus, its concentration might be influenced by pregnancy-related changes other than iodine intake, such as the increase in thyroid secretory activity and the peak in hCG at the end of the first trimester.

Although Tg seems a useful biomarker of iodine nutrition in groups, the use of Tg for an individual is unclear. As Tg concentration seems to reflect changes in iodine intake in the preceding weeks to months, Tg should be validated using multiple urinary-iodine measurements collected prior to the Tg measurement, or a measurement of long-term iodine intake estimated from an FFQ. An RCT in mildly iodine-deficient adults showed that 24 weeks of iodine supplementation were required to observe a fall in Tg from 20 ng/ml at baseline to 13 ng/ml (the proposed cut-off for children) [15]. These findings were supported by an RCT in mildly-to-moderately iodine-deficient pregnant women, where compared to its first-trimester values, by the third trimester, Tg decreased by 27% in response to iodine supplementation, while it increased by 17% in the placebo group [16]. The changes in Tg during pregnancy might be dependent on the baseline iodine status and the amount of iodine stored in the thyroid. For instance, in a previous study of pregnant women, Tg increased from 17 ng/ml at 12 weeks to 20 ng/ml at 35 weeks in the group of women with UI/Creat < 150 µg/g but remained unchanged in the group with UI/Creat ≥ 150 µg/g (~ 16 ng/ml) [30]. Tg also did not differ by trimester in iodine-sufficient euthyroid pregnant women [43].

### Strengths and limitations

Data from a large number of pregnant women residing in areas of iodine sufficiency and mild-to-moderate iodine deficiency enabled the exploration of the relationship between UI/Creat and Tg in two different settings of baseline iodine status. Data on several maternal socio-demographic and socio-economic characteristics were available and accounted for in these analyses, minimising their potential confounding effect on the association between iodine and Tg. The simultaneous measurement of Tg-Abs enabled the exclusion of Tg-Ab positive women thus limiting their known interference with the interpretation of Tg measurements [11].

Only a single Tg measurement from early pregnancy was available; the potential change in Tg over gestation and the association of UI/Creat with Tg in late pregnancy could not be evaluated. The use of single spot-urine samples for the measurement of UIC is a limitation but this was somewhat accounted for by expressing it per unit creatinine; using the UI/Creat instead of UIC alone has lower variability and better reflects the 24-h UIE and serum iodine concentration [5, 7–10]. Information on iodine-supplement use was available only for a sub-set of Generation R women and there were no data on supplement dose hence the effect of iodine-containing supplements on Tg could not be fully evaluated in this cohort.

## Conclusion

Our findings suggest that Tg can be a useful marker of suboptimal iodine nutrition in subgroups of pregnant women residing in areas of overall iodine sufficiency and mild-to-moderate iodine deficiency. However, the ability of Tg to distinguish between overall iodine-sufficient and mildly iodine-deficient populations is not clear and might be confounded by maternal pre-pregnancy thyroidal iodine stores and the pregnancy time-point at which Tg is measured. As more studies are needed into the usefulness of Tg in monitoring the iodine nutritional status in pregnancy, Tg could be used as a complementary functional indicator of iodine status, alongside biomarkers of recent iodine intake such as UIC or UI/Creat. It is important to consider that the measurement of serum-Tg from blood samples is more invasive and costly than measuring UIC from spot-urine samples for epidemiological research and public-health monitoring of population iodine status. Validated biomarkers of individual iodine status are still lacking and require future work.

## Supplementary Information

Below is the link to the electronic supplementary material.

Supplementary file1 (PDF 359 KB)

## Data Availability

Information about how to gain access to the data used is available for Generation R (https://generationr.nl/researchers/) and for INMA (https://www.proyectoinma.org/en/).

## Abbreviations

BMI: Body mass index
CVs: Coefficients of variation
hCG: Human chorionic gonadotropin
INMA: INfancia y Medio Ambiente
IVF: In-vitro fertilisation
LOD: Limit of detection
NIS: Sodium-iodide symporter
OR: Odds ratio
RCT: Randomised controlled trial
Serum-Tg: Serum thyroglobulin concentration
SFFQs: Semi-quantitative food-frequency questionnaires
Tg: Thyroglobulin
Tg-Ab: Thyroglobulin antibodies
TPO-Ab: Thyroid peroxidase antibodies
UI/Creat: Urinary iodine-to-creatinine ratio
UIC: Urinary iodine concentration
24-h UIE: 24-Hour urinary iodine excretion
