# Supplementary material for: Exploration of thyroglobulin as a biomarker of iodine status in iodine-sufficient and mildly iodine-deficient pregnant women
Source: Eur J Nutr. 2023 Mar 27;62(5):2139–54. doi: 10.1007/s00394-023-03131-x (PMC10349736; doi:10.1007/s00394-023-03131-x)
Supplement: Supplementary file 1 — Supplementary file1 (PDF 359 KB) [file 394_2023_3131_MOESM1_ESM.pdf]

European Journal of Nutrition

Electronic Supplementary Material:

Exploration of thyroglobulin as a biomarker of iodine status in iodine-sufficient and mildly iodine-deficient pregnant women

Mariana Dineva, Margaret P. Rayman, Deborah Levie, Sandra Hunziker, Mònica Guxens, Robin P. Peeters, Mario Murcia, Marisa Rebagliato, Amaia Irizar, Alba Jimeno-Romero, Jordie Sunyer, Tim I.M. Korevaar, and Sarah C. Bath[a]

[a]**Corresponding author:** Dr Sarah C. Bath, Department of Nutritional Sciences, Faculty of Health and Medical Sciences, University of Surrey, Guildford GU2 7XH, United Kingdom. E-mail: s.bath@surrey.ac.uk

Table of Contents

Supplementary Table 1 Multiple imputation of missing values 2

Supplementary Table 2 Median (25-75th percentiles) serum thyroglobulin concentration (serum-Tg, ng/ml) according to sample characteristics by cohort 3

Supplementary Table 3 Socio-demographic and socio-economic determinants[a] of serum thyroglobulin concentration in pregnant women by cohort (adjusted analyses) 5

Supplementary Table 4 Dietary determinants[a] of serum thyroglobulin concentration in pregnant women by cohort (adjusted analyses) 7

Supplementary Table 5 Proportion (unadjusted) of pregnant women with elevated serum thyroglobulin concentration (serum-Tg >55 ng/ml[a]) and odds (adjusted) of elevated serum-Tg according to food-group intakes and iodine-containing supplement-use by cohort (sensitivity analyses) 8

Supplementary Table 6 Median (25-75th percentiles) serum thyroglobulin concentration (serum-Tg, ng/ml) in pregnant women according to UI/Creat categories by cohort 9

Supplementary Table 7 Descriptives of UIC and UI/Creat in pregnant women with 'normal' *vs* elevated serum thyroglobulin concentration (serum-Tg >55 ng/ml[a]) and the proportion with elevated serum-Tg by UI/Creat groups in each cohort (sensitivity analyses) 10

Supplementary Table 1 Multiple imputation of missing values

	**Generation R**	**INMA**
Total women in the analyses [a]	3,548	1,168
% (n) women with at least one missing value on any relevant variables (see below)	38.6% (1,370)	10.4% (121)
% (n) missing values in total	7.6% (3,231)	1.9% (245)
Relevant variables with imputed data	9 of 12	11 of 11
Number of imputed values per variable		
Maternal age	32	2
Pre-pregnancy BMI	692	1
Ethnicity	182	5
Parity	32	4
Smoking status	432	77
Alcohol consumption	471	85
Education level	296	6
Net household income	777	N/A
Marital status	317	N/A
Living with a partner	N/A	2
Gestational week at urine sampling	0 [b]	1
Gestational week at blood sampling	0 [b]	2
Child's sex	0 [b]	60

[a] Missing values were identified and multiple imputation was performed for this selected sample.

[b] Data were not imputed due to no missing values for these variables.

Abbreviations: BMI, body mass index; N/A, data not available in this cohort

Supplementary Table 2 Median (25-75th percentiles) serum thyroglobulin concentration (serum-Tg, ng/ml) according to sample characteristics by cohort

	Generation R (n=3,548)			INMA (n=1,168)		
	**n**	**Median (25-75th) Tg**	***P*** [a]	**n**	**Median (25-75th) Tg**	***P*** [a]
**Maternal factors**						
TPO-Ab status			0.179			
Negative	3,060	11.2 (7.1 - 17.0)		N/A	N/A	
Positive	130	9.4 (4.8 - 20.5)		N/A	N/A	
Ethnicity			**<0.001**			0.917
Reference group[b]	1,865	11.4 (7.2 - 17.3)		1,070	11.5 (6.9 - 19.0)	
Non-Dutch:						
Indonesian	95	10.8 (7.1 - 16.0)		N/A	N/A	
Cape Verdean	155	13.6 (7.8 - 18.8)		N/A	N/A	
Moroccan	198	10.1 (6.2 - 14.8)		N/A	N/A	
Dutch Antilles	114	17.1 (10.6 - 27.9)		N/A	N/A	
Surinamese	316	12.1 (7.3 - 18.7)		N/A	N/A	
Turkish	300	8.7 (6.0 - 12.9)		N/A	N/A	
Other, Non-Western	143	10.5 (6.7 - 15.7)		N/A	N/A	
Asian	51	10.1 (7.2 - 15.9)		N/A	N/A	
Other, Western	311	10.5 (6.5 - 16.6)		N/A	N/A	
Non-Spanish:						
Latin-American	N/A	N/A		67	10.9 (7.7 - 17.0)	
European/others	N/A	N/A		31	12.3 (5.9 - 19.8)	
Parity			**0.002**			0.122
0	2,033	11.4 (7.3 - 17.6)		639	11.0 (6.9 - 18.4)	
1	1,040	10.9 (6.9 - 16.3)		446	11.6 (6.9 - 18.7)	
≥ 2	475	10.5 (6.3 - 16.2)		83	13.9 (7.5 - 24.8)	
Smoking status			**<0.001**			**<0.001**
Never smoked	2,524	10.7 (6.7 - 16.2)		836	10.7 (6.3 - 17.4)	
Stopped smoking	337	11.0 (7.3 - 17.8)		160	12.5 (8.0 - 20.9)	
Continued smoking	687	13.0 (8.4 - 19.2)		172	15.8 (9.4 - 26.9)	
Alcohol consumption			**<0.001**			**0.002**
No	1,650	10.5 (6.7 - 16.3)		1,059	11.3 (6.7 - 18.4)	
Yes	1,898	11.5 (7.3 - 17.6)		109	14.8 (8.9 - 23.1)	
**Markers of socio-economic status**						
Education level			0.694			**<0.001**
Low	386	10.9 (6.8 - 16.6)		248	12.9 (8.5 - 23.3)	
Medium	1,612	11.2 (7.0 - 17.4)		464	11.4 (6.9 - 18.5)	
High	1,550	11.1 (7.0 - 17.0)		456	10.8 (6.1 - 17.0)	

(continued on next page)

Supplementary Table 2 Median (25-75th percentiles) serum thyroglobulin concentration (serum-Tg, ng/ml) according to sample characteristics by cohort *(continued)*

	Generation R (n=3,548)			INMA (n=1,168)		
	**n**	**Median (25-75th) Tg**	***P*** [a]	**n**	**Median (25-75th) Tg**	***P*** [a]
**Markers of socio-economic status**						
Net household income (€ per month)			0.799			
Low < €1200	780	11.1 (7.0 - 17.1)		N/A	N/A	
Medium €1200-2200	713	11.1 (7.0 - 17.0)		N/A	N/A	
High > €2200	2,055	11.1 (7.0 - 17.2)		N/A	N/A	
Marital status			**<0.001**			
Married	1,703	10.8 (6.7 - 16.2)		N/A	N/A	
Never-married	1,845	11.4 (7.2 - 18.0)		N/A	N/A	
Living with a partner						**0.013**
Yes	N/A	N/A		1,157	11.4 (6.9 - 18.7)	
No	N/A	N/A		11	20.2 (12.9 - 33.7)	
**Child factors**						
Child's sex			0.923			0.581
Male	1,807	11.1 (7.0 - 17.3)		587	11.5 (6.8 - 18.6)	
Female	1,741	11.1 (7.1 - 16.7)		581	11.5 (7.1 - 19.6)	

[a] *P*-values from a Mann-Whitney U test (for two-categorical variables) or a Kruskal-Wallis test (for variables with more than two categories).

[b] Reference group for ethnicity in Generation R (Reference group=Dutch) and in INMA (Reference group=Spanish). **Abbreviations:** N/A, data not available or not applicable; Tg, thyroglobulin; TPO-Ab, thyroid peroxidase antibody

Supplementary Table 3 Socio-demographic and socio-economic determinants[a] of serum thyroglobulin concentration in pregnant women by cohort (adjusted analyses)

	Generation R (n=3,190)			INMA (n=1,168)		
	**n**	**B (95% CI)**	***P*** [b]	**n**	**B (95% CI)**	***P*** [c]
**Maternal factors**						
Gestational week at blood extraction	3,190	-0.013 (-0.026, 0.001)	0.064	1,168	0.021 (-0.011, 0.054)	0.203
Maternal age, years	3,190	0.006 (-0.000, 0.013)	0.058	1,168	-0.013 (-0.029, 0.002)	0.084
Pre-pregnancy BMI, kg/m^2	3,190	0.001 (-0.006, 0.008)	0.823	1,168	-0.002 (-0.017, 0.012)	0.735
TPO-Ab status						
Negative	3,060	Ref.		N/A	N/A	N/A
Positive	130	-0.127 (-0.258, 0.005)	0.059	N/A	N/A	N/A
Ethnicity						
Reference group[d]	1,690	Ref.		1,070	Ref.	
Non-Dutch:						
Indonesian	85	-0.078 (-0.243, 0.087)	0.355	N/A	N/A	N/A
Cape Verdean	136	0.164 (0.020, 0.307)	**0.026**	N/A	N/A	N/A
Moroccan	175	-0.040 (-0.179, 0.100)	0.578	N/A	N/A	N/A
Dutch Antilles	104	0.404 (0.241, 0.567)	**<0.001**	N/A	N/A	N/A
Surinamese	279	0.099 (-0.007, 0.206)	0.067	N/A	N/A	N/A
Turkish	271	-0.307 (-0.425, -0.188)	**<0.001**	N/A	N/A	N/A
Other, Non-Western	127	-0.062 (-0.206, 0.083)	0.403	N/A	N/A	N/A
Asian	45	-0.019 (-0.212, 0.249)	0.875	N/A	N/A	N/A
Other, Western	278	-0.036 (-0.133, 0.060)	0.457	N/A	N/A	N/A
Non-Spanish:						
Latin-American	N/A	N/A	N/A	67	-0.036 (-0.276, 0.204)	0.770
European/others	N/A	N/A	N/A	31	-0.065 (-0.409, 0.279)	0.711
Parity						
0	1,833	Ref.		639	Ref.	
1	920	-0.082 (-0.144, -0.020)	**0.009**	446	0.065 (-0.060, 0.189)	0.307
≥ 2	437	-0.123 (-0.212, -0.035)	**0.007**	83	0.169 (-0.065, 0.403)	0.157
Smoking status						
Never smoked	2,275	Ref.		836	Ref.	
Stopped smoking	295	0.024 (-0.071, 0.119)	0.620	160	0.197 (0.015, 0.380)	**0.034**
Continued smoking	620	0.248 (0.174, 0.323)	**<0.001**	172	0.385 (0.210, 0.560)	**<0.001**
Alcohol consumption						
No	1,476	Ref.		1,059	Ref.	
Yes	1,714	0.026 (-0.040, 0.091)	0.442	109	0.205 (0.001, 0.410)	**0.049**
**Markers of socio-economic status**						
Education level						
Low	343	Ref.		248	Ref.	
Medium	1,443	-0.012 (-0.113, 0.089)	0.815	464	-0.234 (-0.384, -0.083)	**0.002**
High	1,404	-0.019 (-0.131, 0.094)	0.746	456	-0.228 (-0.385, -0.017)	**0.004**

(continued on next page)

Supplementary Table 3 Socio-demographic and socio-economic determinants[a] of serum thyroglobulin concentration in pregnant women by cohort (adjusted analyses) *(continued)*

	Generation R (n=3,190)			INMA (n=1,168)		
	**n**	**B (95% CI)**	***P*** [b]	**n**	**B (95% CI)**	***P*** [c]
**Markers of socio-economic status** *(continued)*						
Net household income (€ per month)						
Low < €1200	689	Ref.		N/A	N/A	N/A
Medium €1200-2200	636	0.027 (-0.077, 0.132)	0.606	N/A	N/A	N/A
High > €2200	1,865	-0.001 (-0.106, 0.104)	0.987	N/A	N/A	N/A
Marital status						
Married	1,539	Ref.		N/A	N/A	N/A
Never-married	1,651	-0.041 (-0.102, 0.021)	0.194	N/A	N/A	N/A
Living with a partner						
Yes	N/A	N/A	N/A	1,157	Ref.	
No	N/A	N/A	N/A	11	0.361 (-0.213, 0.935)	0.217
**Child factors**						
Child's sex						
Male	1,625	Ref.		587	Ref.	
Female	1,565	-0.001 (-0.053, 0.051)	0.971	581	-0.003 (-0.119, 0.112)	0.958

[a] Effect estimates (B=unstandardised regression coefficients), their 95% CIs and *P*-values are from multiple linear regression models performed for each cohort with (natural) log-transformed thyroglobulin (Tg) concentration as the dependent variable and maternal characteristics as independent variables (for full models, see footnotes b and c). Reported B coefficients represent the change in the mean (natural) log of Tg per unit increase in the continuous independent variables and for each category compared to the reference for the categorical independent variables.

[b] Generation R Model (R^2=0.038, $P < 0.0001$): gestational week at blood extraction, age (years), pre-pregnancy BMI (kg/m^2), TPO-Ab status, ethnicity, parity, smoking status, alcohol consumption, education, net household income, marital status and child's sex.

[c] INMA Model (R^2=0.043, $P < 0.0001$): gestational week at blood extraction, age (years), pre-pregnancy BMI (kg/m^2), ethnicity, parity, smoking status, alcohol consumption, education, living with a partner and child's sex.

[d] Reference group of ethnicity in Generation R (Reference group=Dutch) and in INMA (Reference group=Spanish).

Abbreviations: BMI, body mass index; 95% CI, confidence interval; N/A, data not available or not applicable; Ref., reference category; Tg, thyroglobulin; TPO-Ab, thyroid peroxidase antibody

Supplementary Table 4 Dietary determinants[a] of serum thyroglobulin concentration in pregnant women by cohort (adjusted analyses)

Food group intake (100 g/day)	Generation R (n=2,768)		INMA (n=1,155)	
	**B (95% CI)**	***P*** [b]	**B (95% CI)**	***P*** [c]
Vegetables	0.58 (0.04, 1.15)	**0.037**	-0.11 (-0.79, 0.61)	0.751
Fruit	-0.16 (-0.48, 0.16)	0.322	0.23 (-0.18, 0.66)	0.272
Nuts and seeds	-1.44 (-4.09, 2.14)	0.388	6.75 (-3.02, 27.06)	0.234
Potatoes	-0.57 (-1.37, 0.30)	0.196	-0.35 (-2.14, 1.76)	0.727
Legumes	-1.06 (-4.46, 4.00)	0.632	-1.62 (-3.92, 1.33)	0.256
Cereals and cereal products	-0.20 (-0.87, 0.50)	0.562	0.49 (-0.91, 2.06)	0.512
Cakes, confectionery and added sugar	-0.46 (-1.38, 0.54)	0.356	0.99 (-1.80, 4.54)	0.520
Added fats[d]	-0.01 (-0.04, 0.02)	0.454	0.03 (-0.04, 0.10)	0.343
Milk and dairy products	-0.20 (-0.38, -0.02)	**0.030**	-0.08 (-0.47, 0.33)	0.701
Meat and meat products	0.38 (-0.56, 1.40)	0.440	1.33 (-0.76, 3.82)	0.227
Eggs	-1.24 (-4.03, 2.59)	0.481	6.51 (-2.94, 25.74)	0.234
Fish and shellfish	-0.13 (-2.47, 2.81)	0.920	0.41 (-1.70, 2.94)	0.726
Condiments and seasoning[d]	-0.06 (-0.14, 0.01)	0.105	-1.40 (-2.82, 0.24)	0.091
Processed and fried foods	-0.05 (-0.44, 0.36)	0.815	-0.60 (-1.56, 0.45)	0.256
Non-alcoholic beverages	-0.01 (-0.08, 0.06)	0.696	0.02 (-0.13, 0.18)	0.787
Alcoholic beverages	-0.22 (-1.72, 1.50)	0.787	-0.53 (-4.20, 4.85)	0.818
Miscellaneous	-1.63 (-3.12, 0.13)	0.068	N/A	N/A

[a] Effect estimates (B=unstandardised regression coefficients), their 95% CIs and *P*-values are from multiple linear regression models performed for each cohort with (natural) log-transformed thyroglobulin (Tg) concentration as the dependent variable and maternal food-group intakes as independent variables, adjusted for confounders (see footnotes b and c). Reported B coefficients represent the actual change in the geometric mean of Tg (ng/ml) associated with a 100-g increase in intake of a food group (exceptions, see footnote d). B coefficients and their 95% CIs were calculated by back-transformation from logarithmic scale as described previously [**1**]. Values are adjusted for dietary intake of other food groups, energy intake and other potential confounders (for full models, see footnotes b and c).

[b] Generation R Model (R^2=0.032, $P < 0.0001$): all food-group intakes (g/day) + energy (kcal/day) + gestational week at blood extraction, age (years), pre-pregnancy BMI (kg/m^2), TPO-Ab status, ethnicity, parity, smoking status, alcohol consumption, education, net household income, marital status and child's sex.

[c] INMA Model (R^2=0.040, $P < 0.0001$): all food-group intakes (g/day) + energy (kcal/day) + gestational week at blood extraction, age (years), pre-pregnancy BMI (kg/m^2), ethnicity, parity, smoking status, alcohol consumption, education, living with a partner and child's sex.

[d] Effect size is expressed per 1 g (instead of per 100 g) for added fats and condiments.

Abbreviations: BMI, body mass index; 95% CI, confidence interval; N/A, data not available or not applicable; Tg, thyroglobulin; TPO-Ab, thyroid peroxidase antibody

References:

1. Dineva M, Rayman MP, Levie D, et al (2019) Similarities and differences of dietary and 707 other determinants of iodine status in pregnant women from three European birth cohorts. Eur 708 J Nutr 59:371–387. https://doi.org/10.1007/s00394-019-01913-w

Supplementary Table 5 Proportion (unadjusted) of pregnant women with elevated serum thyroglobulin concentration (serum-Tg >55 ng/ml[a]) and odds (adjusted) of elevated serum-Tg according to food-group intakes and iodine-containing supplement-use by cohort (sensitivity analyses)

	**Generation R (n=2,768)**						**INMA (n=1,155)**					
	**Unadjusted**			**Adjusted**			**Unadjusted**			**Adjusted**		
	**n**	**n (%)**	***P*** [b]	**n**	**OR (95% CI)**	***P*** [c]	**n**	**n (%)**	***P*** [b]	**n**	**OR (95% CI)**	***P*** [c]
**Milk intake**			0.062[d]						**0.003**			
None	148	6 (4.1%)		135	Ref		111	7 (6.3%)		111	Ref	
≤ 1 glass (≤ 200 g/day)	1,506	23 (1.5%)		1,345	0.430 (0.153, 1.203)	0.108	615	10 (1.6%)		615	0.215 (0.076, 0.608)	**0.004**
> 1 glass (> 200 g/day)	1,114	15 (1.3%)		1,002	0.308 (0.097, 0.975)	**0.045**	429	6 (1.4%)		429	0.134 (0.038, 0.472)	**0.002**
**Fish and shellfish intake**			0.163						1.000			
None (0 g/day)	555	13 (2.3%)		500	Ref		N/A	N/A			N/A	N/A
Some (> 0 g/day)	2,213	31 (1.4%)		1,982	0.570 (0.267, 1.218)	0.147	N/A	N/A			N/A	N/A
≤ 1 portion (≤ 120 g/day)	N/A	N/A		N/A	N/A	N/A	1,041	21 (2.0 %)		1,041	Ref	
> 1 portion (> 120 g/day)	N/A	N/A		N/A	N/A	N/A	114	2 (1.8 %)		114	1.011 (0.215, 4.745)	0.989
**Bread intake**			0.877						N/A			
≤ median (≤ 98 g/day)	1,378	21 (1.5%)		1,221	Ref		N/A	N/A		N/A	N/A	N/A
> median (> 98 g/day)	1,377	23 (1.7%)		1,249	1.510 (0.679, 3.360)	0.312	N/A	N/A		N/A	N/A	N/A
**Salt intake (inc. iodised)**			N/A						0.356			
None (0 g/day)	N/A	N/A		N/A	N/A	N/A	374	10 (2.7%)		374	Ref	
Some (> 0 g/day)	N/A	N/A		N/A	N/A	N/A	781	13 (1.7%)		781	0.593 (0.250, 1.408)	0.236
**Iodine-supplement use**[e]			N/A						0.144			
No	N/A	N/A		N/A	N/A	N/A	631	17 (2.7%)		631	Ref	
Yes	N/A	N/A		N/A	N/A	N/A	537	7 (1.3%)		537	0.570 (0.223, 1.457)	0.240

[a] Cut-off value considered elevated from the laboratory where the Tg measurements were performed (see 'Methods').

[b] *P*-values from a Chi-square test (after Continuity Correction for comparisons in 2x2 tables).

[c] *P*-values from multiple logistic regression models with elevated serum-Tg as the dependent variable (categorised as >55 ng/ml). In Generation R: 1) models with food-group intakes were adjusted for energy intake, gestational week at blood extraction, age, pre-pregnancy BMI, TPO-Ab status, ethnicity, parity, smoking status, alcohol consumption, education, net household income, marital status, and child's sex; and 2) models with iodine-supplement use were not performed (see footnote 'e'). In INMA: 1) models with food-group intakes were adjusted for energy intake, gestational week at blood extraction, age, pre-pregnancy BMI, ethnicity, parity, smoking status, alcohol consumption, education, living with a partner, and child's sex; and 2) models with iodine-supplement use were adjusted for gestational week at blood extraction, age, pre-pregnancy BMI, ethnicity, parity, smoking status, alcohol consumption, and education.

[d] Fisher's Exact Test *P*-value was reported due to some cells (>20%) with expected count < 5.

[e] Data on iodine-supplement use were available only for a sub-sample in Generation R (see 'Methods') and statistical analyses with elevated serum-Tg (i.e., categorical outcome) were not performed in this cohort due to very small numbers; e.g., there was only 1 woman who was an iodine-supplement non-user and had elevated serum-Tg for the models with Tg >55 ng/ml.

Abbreviations: BMI, body mass index; 95% CI, confidence interval; N/A, data not available or not applicable; OR, odds ratio; Ref., reference category; Tg, thyroglobulin; TPO-Ab, thyroid peroxidase antibody

Supplementary Table 6 Median (25-75th percentiles) serum thyroglobulin concentration (serum-Tg, ng/ml) in pregnant women according to UI/Creat categories by cohort

	Generation R (n=1,071)			INMA (n=1,000)		
	**n**	**Median (25-75th) Tg**	***P*** [a]	**n**	**Median (25-75th) Tg**	***P*** [a]
UI/Creat grouping 1			**0.010**			**<0.001**
< 150 µg/g	311	12.0 (7.4 - 17.9)		513	12.8 (7.8 - 21.7)	
≥ 150 µg/g	760	10.4 (6.5 - 16.4)		487	10.4 (6.2 - 16.1)	
UI/Creat grouping 2			**0.028**			**<0.001**
< 100 µg/g	113	13.2 (7.7 - 19.9)		284	13.9 (8.3 - 21.7)	
100 - 149 µg/g	198	11.6 (7.2 - 16.6)		229	11.9 (6.9 - 21.2)	
150 - 249 µg/g	340	10.2 (6.5 - 16.1)		245	10.6 (6.1 - 17.4)	
≥ 250 µg/g	420	10.6 (6.5 - 16.7)		242	10.1 (6.4 - 15.4)	

[a] *P*-values from a Mann-Whitney U test (for two-categorical variables) or a Kruskal-Wallis test (for variables with more than two categories).

Abbreviations: Tg, thyroglobulin; UI/Creat, urinary iodine-to-creatinine ratio

Supplementary Table 7 Descriptives of UIC and UI/Creat in pregnant women with ‘normal’ *vs* elevated serum thyroglobulin concentration (serum-Tg >55 ng/ml[a]) and the proportion with elevated serum-Tg by UI/Creat groups in each cohort (sensitivity analyses)

	**Generation R (n=1,071)**					**INMA (n=1,000)**				
	**‘Normal’ Tg**		**Elevated Tg**			**‘Normal’ Tg**		**Elevated Tg**		
**Cut-off > 55 ng/ml [a]**	**n**	**Tg ≤ 55 ng/ml**	**n**	**Tg > 55 ng/ml**	***P* [b]**	**n**	**Tg ≤ 55 ng/ml**	**n**	**Tg > 55 ng/ml**	***P* [b]**
UIC, µg/L, median (25-75th percentiles)	1,058	167 (97 - 285)	13	141 (98 - 292)	0.624	981	132 (76 - 223)	19	97 (55 - 135)	**0.027**
UI/Creat, µg/g, median (25-75th percentiles)	1,058	209 (139 - 307)	13	188 (112 - 302)	0.374	981	148 (95 - 247)	19	102 (66 - 142)	**0.012**
**N (%) with ‘normal’ and elevated Tg**					0.539[c]					**0.028**
UI/Creat < 150 µg/g	311	306 (98.4%)		5 (1.6%)		513	498 (97.1%)		15 (2.9%)	
UI/Creat ≥ 150 µg/g	760	752 (98.9%)		8 (1.1%)		487	483 (99.2%)		4 (0.8%)	

[a] Cut-off value considered elevated from the laboratory where the Tg measurements were performed (see ‘Methods’). For the results with a cut-off for elevated Tg >40 ng/ml (based on previous studies in adults and school-aged children), see Table 5 in the main results.

[b] *P*-values from a Mann-Whitney U test or a Chi-square test (after Continuity Correction for comparisons in 2x2 tables).

[c] Fisher's Exact Test *P*-value reported due to some cells with expected count < 5.

Abbreviations: Tg, thyroglobulin; UI/Creat, urinary iodine-to-creatinine ratio; UIC, urinary iodine concentration
